# EPO Mediates Neurotrophic, Neuroprotective, Anti-Oxidant, and Anti-Apoptotic Effects via Downregulation of miR-451 and miR-885-5p in SH-SY5Y Neuron-Like Cells

**DOI:** 10.3389/fimmu.2014.00475

**Published:** 2014-09-30

**Authors:** Begum Alural, Gizem Ayna Duran, Kemal Ugur Tufekci, Jens Allmer, Zeynep Onkal, Dogan Tunali, Kursad Genc, Sermin Genc

**Affiliations:** ^1^Advanced Biomedical Research Center, Dokuz Eylul University, Izmir, Turkey; ^2^Department of Neuroscience, Health Science Institute, Dokuz Eylul University, Izmir, Turkey; ^3^Department of Molecular Biology and Genetics, Izmir Institute of Technology, Urla, Turkey

**Keywords:** erythropoietin, miRNAs, cell death, proliferation, apoptosis, migration, neurite outgrowth, oxidative stress

## Abstract

Erythropoietin (EPO) is a neuroprotective cytokine, which has been applied in several animal models presenting neurological disorders. One of the proposed modes of action resulting in neuroprotection is post-transcriptional gene expression regulation. This directly brings to mind microRNAs (miRNAs), which are small non-coding RNAs that regulate gene expression at the post-transcriptional level. It has not yet been evaluated whether miRNAs participate in the biological effects of EPO or whether it, inversely, modulates specific miRNAs in neuronal cells. In this study, we employed miRNA and mRNA arrays to identify how EPO exerts its biological function. Notably, miR-451 and miR-885-5p are downregulated in EPO-treated SH-SY5Y neuronal-like cells. Accordingly, target prediction and transcriptome analysis of cells treated with EPO revealed an alteration of the expression of genes involved in apoptosis, cell survival, proliferation, and migration. Low expression of miRNAs in SH-SY5Y was correlated with high expression of their target genes, vascular endothelial growth factor A, matrix metallo peptidase 9 (*MMP9)*, cyclin-dependent kinase 2 (*CDK2)*, erythropoietin receptor, Mini chromosome maintenance complex 5 (*MCM5)*, B-cell lymphoma 2 (*BCL2*), and Galanin (*GAL)*. Cell viability, apoptosis, proliferation, and migration assays were carried out for functional analysis after transfection with miRNA mimics, which inhibited some biological actions of EPO such as neuroprotection, anti-oxidation, anti-apoptosis, and migratory effects. In this study, we report for the first time that EPO downregulates the expression of miRNAs (miR-451 and miR-885-5p) in SH-SY5Y neuronal-like cells. The correlation between the over-expression of miRNAs and the decrease in EPO-mediated biological effects suggests that miR-451 and miR-885-5p may play a key role in the mediation of biological function.

## Introduction

Erythropoietin (EPO) is a hematopoietic cytokine that regulates erythropoiesis (1). Its primary production sites are the fetal liver and the adult kidney. Nonetheless, EPO is also produced by many non-hematopoietic tissues including the central nervous system (CNS). It has now been well established that different cell types of the CNS (neurons, glial cells, and endothelial cells) produce EPO (2). In addition to producing EPO, these cells also express the erythropoietin receptor (EPOR) on their plasma membranes thus underlining the crucial role that EPO plays in the physiological function of these cells (3).

Recombinant human erythropoietin (rhEPO) is a well-known FDA approved drug that has been used for the treatment of chronic anemia associated with renal failure, cancer, and prematurity since 1989 (1). It has been shown that EPO can cross the blood–brain barrier (BBB) via a receptor-mediated mechanism (4). EPO exerts remarkable neuroprotection for both, *in vitro* and *in vivo* models of nervous system disorders. *In vitro* studies showed that EPO is directly neuroprotective in hypoxic, hypoglycemic, and excitotoxic neuronal injury models (5). Exogenous administration of rhEPO protects the brain against neurodegeneration in a wide variety of experimental neurological disorders including stroke, neonatal hypoxic–ischemic encephalopathy, multiple sclerosis (MS), subarachnoid hemorrhage, traumatic brain injury, epileptic seizure, Parkinson’s disease (PD), Alzheimer’s disease (AD), and spinal cord injury (2, 6). EPO is currently being under investigation in several clinical trials regarding neuropsychiatric diseases (6).

Erythropoietin protects the CNS cells by limiting the production of tissue-injuring molecules such as reactive oxygen species (ROS) and glutamate, attenuation of apoptosis, modulation of inflammation, stimulation of angiogenesis, and induction of neurogenesis (7). However, the tissue-protective mechanisms of EPO are still not fully understood. EPO’s impact on regulation at both the transcriptional and the post-transcriptional levels may play a critical role for its cellular effects. EPO activates various signaling pathways that result in gene expression changes responsible for its biological activities (8). Previous *in vitro* (9) and *in vivo* (10–14) studies using mRNA microarrays evaluated genome-wide expression changes induced by EPO.

Some genes regulated by EPO may host microRNAs (miRNAs), which are then automatically regulated as well. This is then further propagated to the miRNA targets whose transcriptions generally increase with downregulation of its associated miRNAs. miRNAs are short, single stranded, RNA molecules that regulate gene expression at the post*-*transcriptional level (15). Since their discovery about two decades ago (16), they have spawned a great amount of interest and, thus the modulation of gene expression by miRNAs has been highly investigated. These small RNA molecules generally downregulate gene expression usually by binding to complementary sequences in their target mRNAs’ 3′ untranslated region. miRNAs are likely to be involved in most biological processes including development, cell cycle, apoptosis, proliferation, and angiogenesis (15). They have been demonstrated to be essential for brain development and function, and to influence the neuropsychiatric diseases (17). Therefore, it is reasonable to hypothesize that some miRNAs play roles in EPO-related neuroprotective mechanisms in neuronal cells. To date, there is only one study that indicates the importance of miRNAs for the biological effects of EPO on erythropoiesis (18). However, there is currently no study that demonstrates the contribution of miRNAs to the EPO-mediated neuroprotective mechanisms in neuronal cells.

The aim of this study is to determine global expression changes of miRNAs induced by EPO *in vitro* and the role of specific miRNAs in biological function of EPO in SH-SY5Y neuron-like cells. To achieve this, we have identified specific miRNAs via microarray analysis whose transcriptional levels were further validated by quantitative PCR (qPCR). EPO caused changes in the expression of specific miRNAs and their target genes involved in cell proliferation, migration, cell survival, and redox regulation. Moreover, these results were confirmed with functional studies by using miRNA mimics. Our results provide a new molecular insight into the cellular and molecular mechanism of EPO action in neuronal cells.

## Materials and Methods

### Cell culture and treatment

SH-SY5Y cells were maintained in Dulbecco’s Modified Eagle Medium: nutrient mixture F-12 (DMEM:F12) (Gibco, Gaithersburg, MD, USA) supplemented with heat-inactivated fetal bovine serum (10% v/v), l-glutamine (1% v/v), and penicillin-streptomycin (1% v/v) at 37°C in 5% CO_2_.

Twenty-four hours after initial plating of the SH-SY5Y cells, EPO was added at 1 U/ml concentration. Samples were collected after 24 h for quantification of miRNAs expression.

### MicroRNA extraction and microarray screening

Total RNA was isolated from EPO-treated and -untreated cells using the miRNeasy kit (Qiagen GmbH, Hilden) according to the manufacturer’s protocol. Microarray studies were carried out by FEBIT Company as described before (19). Samples were analyzed with a Geniom Real time Analyzer (GRTA, FEBIT GmbH, Heidelberg, Germany) using the Geniom miRNA Homo sapiens Biochip. Every array included 710 miRNAs and miRNA star sequences (seven replicates) which are annotated in the Sanger miRBase 20.0. The labeling of samples with biotin was performed by microfluidic-based enzymatic on-chip labeling of miRNAs. Hybridization was carried out for 16 h at 42°C, and the biochip was washed automatically. The image data were analyzed with the Geniom Wizard Software. The analyses included data background correction, normalization, and determination of differentially expressed miRNAs. Background correction was performed by subtracting the median of blank controls from median of each spots. Quantile normalization and variance stabilizing normalization were applied for normalization of the data across different arrays. The significant differentially expressed miRNAs were determined by an adjusted *p* value lower than 0.05 based on the multiple testing using the Benjamini–Hochberg procedures (20, 21).

### Real-Time PCR analysis of miR-451 and miR-885-5p

Quantitative PCR assay was used to confirm the miRNAs (miR-451 and miR-885-5p) that were found to be differentially regulated in the microarray experiment. Total RNA was isolated from cells using Qiagen miRNeasy Mini Kit and RNA quantity was measured with the NanoDrop Spectrophotometer (Thermo Scientific, USA). Then, 2 μg RNA was reverse-transcribed with a specific stem-loop primer using miScript II RT Kit (Qiagen, Valencia, CA, USA). Real-time PCR was performed using miScript SYBR Green PCR Kit on a Lightcycler 1.5 Real-Time PCR System (Roche Diagnostics, Germany). Primers for mature miR-451, miR-885-5p SNORD95, and U6 were purchased from Qiagen (Valencia, CA, USA). The PCR reactions were performed at 95°C for 10 min, followed by 40 cycles at 95°C for 15 s and at 60°C for 30 s. The specificity of the PCR products was analyzed by the melting curve analysis. The relative expression levels of miRNAs were normalized to the average amounts of U6 and SNORD95 snRNA using the 2^−ΔΔCt^ formulation (22).

### MicroRNA target prediction

miRWalk online prediction software^1^ was used for searching the validated and predicted targets of the altered miRNAs. miRWalk combines nine established target prediction tools: miRWalk, Diana-microT, miRanda, miRDB, PICTAR), PITA, RNA22, RNAhybrid, and Targetscan. Target prediction was performed separately for miR-451 and miR-885-5p with a *p* value <0.05. To establish the interaction network of the miRNAs with the Reactome^2^ pathways, only targets found in miRTarBase^3^ and Tarbase^4^ were accepted since they have experimental evidence.

### mRNA array

Microarray analysis was performed by Aros Applied Biotechnology (Denmark) using with the Human-6 v2 Expression BeadChip (Illumina Inc., San Diego, CA, USA). Briefly, total RNA isolated from SH-SY5Y cells treated with or without EPO, by using Qiagen miRNeasy Mini Kit. cDNA was synthesized from 500 ng total RNA, and then hybridized onto an Illumina Beadchip for 16 h at 58°C, then washed, stained, and scanned. Variance stabilization was performed using variance stabilization transformation and the data were normalized by quantile normalization. All the Illumina IDs (probes) were assumed to be differentially expressed if their ANOVA values were ≤0.5. All experiments were performed in triplicate and overall >98% correlation was observed between replicates.

### Real-time qPCR array

Real-time qPCR array analysis was performed by Aros Applied Biotechnology (Denmark) using same RNA samples in microarray experiments for 26 differentially regulated genes in microarray analysis. Briefly, total RNA isolated from SH-SY5Y cells treated with or without EPO, by using Qiagen miRNeasy Mini Kit. cDNA was synthesized from 500 ng total RNA, then real-time PCR array analysis was performed in a total volume of 20 μl including 2 μl of cDNA, primers, and 10 μl of SYBR Green mix. Reactions were run on a Light cycler 480 using the universal thermal cycling parameters. Expression of target mRNA was normalized with respect to actin using the 2^−ΔΔCt^ method.

### Real-time PCR analysis of mRNAs

Total RNA was isolated using the Nucleospin RNA II Kit (Macherey-Nagel, Germany) and converted to cDNA with RevertAid First Strand cDNA Synthesis Kit (Thermo Fermentas, ABD). Real-time qPCR was performed using the Lightcycler 1.5 Instrument (Roche, ABD) and the SYBR-Green I kit, following the manufacturer’s instructions. The primers used in the qPCR reactions are listed in Table 1. PCR amplification of the template cDNAs were performed using following conditions for 40 cycles. After initial denaturation at 95°C for 10 min, temperature cycling of denaturation at 95°C for 10 s, annealing at 60°C for 10 s, and extension at 72°C for 20 s was performed. The specificity of the PCR products was analyzed by the melting curve analysis. The relative expression levels of mRNAs were quantified using the 2^−ΔΔ^ Ct method with endogenous normalization to the average amounts of glyceraldehyde 3-phosphate dehydrogenase (*GAPDH*) and ribosomal protein, large, P0 (*RPLP0*).

**Table 1:** **Primer sequences for RT-qPCR**.

Gene	Primer sequences
GAPDH	Forward 5′-ACCACAGTCCATGCCATCAC-3′
	Reverse 5′-TCCACCACCCTGTTGCTGTA-3′
RPLP0	Forward 5′-GCATCAGTACCCCATTCTATCA-3′
	Reverse 5′-AAGGTGTAATCCGTCTCCACAGA-3′
BCL2	Forward 5′-CTGGTGGACAACATCGCTCTG-3′
	Reverse 5′-GGTCTGCTGACCTCACTTGTG-3′
VEGFA	Forward 5′-TGGTTGCCCTTTTCTACTTTG-3′
	Reverse 5′-GAAGTAGGAAAGGAGGCCATC-3′
CDK2	Forward 5′-ACCACAGTCCATGCCATCAC-3′
	Reverse 5′-TCCACCACCCTGTTGCTGTA-3′
MMP9	Forward 5′-GACCATAGAGGTGCCGGATG-3′
	Reverse 5′-GGACAAGCTCTTCGGCTTCT-3′
MCM5	Forward 5′-AGCAGAAATACCCGGAGCAC-3′
	Reverse 5′-AACGGATGCCAAAAGCACAC-3′
EPOR	Forward 5′-CAATGAAGTAGTGCTCCTAGACG-3′
	Reverse 5′-TCCAGCACCAGATAGGTATCC-3′
GAL	Forward 5′-CGCAGCTCAAGGTACTACTG-3′
	Reverse 5′-GGGGCTGTTGATACACTCGG-3′

### WST-8 assay

Cell viability was assessed by the ability of viable cells to reduce WST-8 reagent [2-(2-methoxy-4-nitrophenyl)-3-(4-nitrophenyl)-5-(2,4-disulfophenyl)-2H-tetrazolium, monosodium salt] to the highly water soluble formazan dye. Cells were seeded in 96-well plate at a density of 2 × 10^4^ cells per well. After treatment with EPO and/or a toxic agent, 10 μl of WST-8 reagent was added to each well containing 100 μl of medium. The plate was then incubated for 4 h at 37°C and the absorbance of each well was measured on a microplate reader (Biotek, USA) at 450 nm with a reference wavelength of 630 nm. The relative cell viability was calculated as the percentage of untreated cells.

### Trypan blue staining

Cell viability was also evaluated by trypan blue dye exclusion. Cells were seeded on a 24-well plate at a density of 5 × 10^5^ cells per well. Following incubation, cells were stained with trypan blue stain for 1 min and fixed with 2% paraformaldehyde for 20 min. Stained cells were photographed by using an inverted microscope (Olympus CKX41, Japan).

### DNA fragmentation assay

The Cell Death Detection ELISA Kit was used to evaluate DNA fragmentation in the cytoplasm of apoptotic cells. SH-SY5Y cells were seeded in a 96-well plate at a density of 2 × 10^4^ cells per well. SH-SY5Y cells were pretreated with EPO (1 U/ml) for 24 h. Then toxic agents were added to the cell culture medium. Staurosporine (STS) was used at a concentration of 25 nM for 6 h; CoCl_2_ was used at a concentration of 250 μM for 24 h. Following treatment, cells were incubated in the lysis buffer for 30 min. Thereafter, the samples were centrifuged at 200×*g* for 10 min and 20 μl of the supernatant was transferred to a streptavidin coated microplate. Then the HRP-conjugated antibody cocktail was added to each well. After 2 h of incubation at room temperature, ABTS (2,2-azino-di-3-ethylbenzthiazoline sulfonate) was added to the wells. Subsequent to 15-min incubation, the plates were read at a wavelength of 405 nm in a microplate reader. The results are represented as fold increase in respect to optical density as compared with control cells.

### Annexin-V Immunostaining

Annexin-V-FITC apoptosis detection kit I (Becton Dickinson, USA) was used to detect apoptotic cells. At the end of incubation, cells were stained with Annexin-V according to the manufacturer’s instructions. After staining procedure, cells were fixed with 4% paraformaldehyde for 20 min. Stained cells were photographed by using a fluorescence microscope (Olympus BX50, Japan).

### Cell proliferation assay

SH-SY5Y cells were seeded in a 24-well plate at a density of 75 × 10^3^ cells per well and treated with or without EPO. After 24 h of EPO treatment, cell culture plates were first washed three times with PBS, the cells then harvested by trypsinization and the cell number was determined using a hemocytometer.

### Intracellular ROS quantification

Intracellular ROS quantification was measured by 2′,7′-dichlorodihydrofluorescein, acetyl ester (CM-H_2_DCFDA) (Invitrogen). Cells were seeded into black 96-well plate at 2 × 10^4^ cells per well and were treated with EPO for 24 h. Then, 100 μl PBS containing 10 μM CM-H_2_DCFDA was added to each well and incubated for 1 h. Afterward, CM-H_2_DCFDA was removed from each well and cells were treated with CoCl_2_ (250 μM) or both CoCl_2_ (250 μM) and EPO for 24 h. Fluorescence levels were determined following excitation with a wavelength of 492 nm at an emission wavelength of 527 nm by using a microplate reader (BioTek, USA).

### Migration assay

The SH-SY5Y cells were seeded at a density of 2 × 10^5^ cells/well in 24-well plates, and incubated in DMEM/F12 containing 10% FBS for 24 h to confluence. A confluent monolayer of each well was scratched with a 200 μl pipette tip and cells were washed twice with PBS and replaced with fresh growth medium containing 1% FBS, with or without EPO. Immediately after the scratch (0 h) and at 24 h, four wounded area were marked in each well and images obtained using phase-contrast inverted microscope (Olympus, CKX41; 10×). The same wounded area was selected for the measurements at each time of study. The number of cells that migrated over the margins of the wounds was counted by using ImageJ 1.42 ^5^. All experiments were performed in triplicate, and each experiment was repeated at least three times.

### Neurit outgrowth and count

SH-SY5Y cells were transfected with miR-451 and miR-885-5p mimics and negative control oligomers as described before (Qiagen, USA). After transfection, cells were treated with 1 U/ml Epo for 24 h, then, cells were photographed by using an inverted microscope (Eclipse TS120, Nikon) using phase-contrast objectives with a 40× magnification.

For quantifying the number of neurites per cell, neurite-bearing cells were counted from at least three randomly selected microscopic fields with an average of 35 cells per field.

Additionally, neurite length was measured from all neurite-bearing cells and the length of each neurite was quantified by using ImageJ 1.42^5^. Neurite lengths that counted from three different images and at least 30 neurites per condition were quantified. Independent cell culture experiments were conducted in triplicate.

### Transfection of miRNAs mimics

MicroRNA mimics and negative controls were purchased from Qiagen. SH-SY5Y cells were transfected 24 h after seeding in cell culture plates. Transfection of cells with miR-451 and miR-885-5p mimics and negative control oligomers (Qiagen) was performed using the HiPerFect transfection reagent (Qiagen) according to the manufacturer’s protocol. The final concentration of the mimics was 50 nM. Samples were collected after 48 h of miRNA mimic transfections for quantification of miRNA and target gene expression.

### Statistical analysis

Statistical analyses were performed with SPSS 18.0. Values represent the average ± SEM. Differences among averages were analyzed by Student’s *t*-test or Mann–Whitney *U* test. *p* values smaller than 0.05 were considered to be statistically significant. The correlation between the results of mRNA array and qPCR array was investigated by Pearson’s correlation analysis test.

## Results

### Profiling microRNA expression changes following EPO treatment

MicroRNA microarrays were used to identify miRNAs with altered expression due to treatment with EPO. SH-SY5Y cells were treated with 1 U/ml EPO for 24 h. Analysis of the microRNA expression using microRNA arrays showed that miR-451 and miR-885-5p expression decreased in response to treatment with EPO in SHSY-5Y cells according to the criteria of a fold change ≤−1.5 or ≥1.5 (*P* < 0.05) (Table S1 in Supplementary Material).

**Figure 1:**
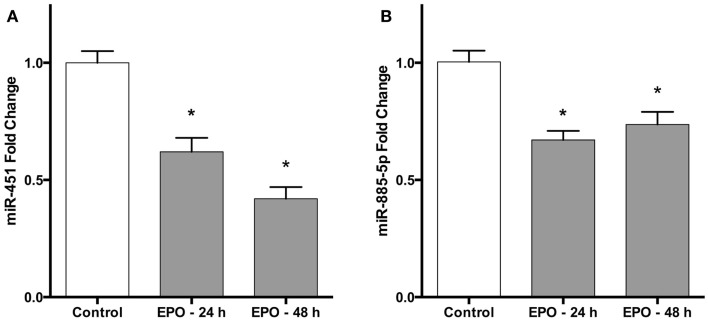
**Erythropoietin decreases miR-451 and miR-885-5p expression in SH-SY5Y cells**. Cells were treated with 1 U/ml EPO during 24 and 48 h. Expression levels of miR-451 **(A)** and miR-885-5p **(B)** in both treated and non-treated cells were quantified by qRT-PCR. EPO; erythropoietin. The data are presented as mean ± SE, *n* = 5 (*p < 0.05).

These results were validated by quantitative qPCR, using miR-451 and miR-885-5p specific primers, which recognize their mature forms. Consistent with the microarray results, both miR-451 and miR-885-5p were downregulated following 24 h of treatment with EPO (Figures 1A,B). In addition, 48 h EPO treatment also decreases expression of miR-451 and miR-885-5p in SH-SY5Y cells (Figures 1A,B).

### Target gene prediction

To search the validated and predicted targets for differentially expressed miRNAs miRWalk software^1^ was used. An mRNA was considered a potential target if it appeared in at least three of the algorithms utilized. Employing that criterion, we obtained 1831 predicted and 117 validated target genes for miR-451 and miR-885-5p.

**Figure 2:**
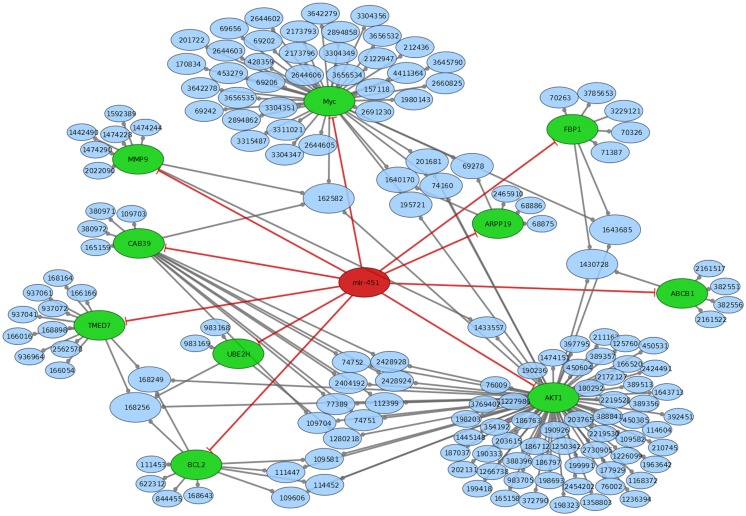
**Mir-451 comes from a link RNA (not shown in graph)**. Mir-451 has 10 known targets (green). These targets are part of a number of reactome pathways (blue). The number in the blue nodes can be used to access the associated reactome pathway, and Table S3 in Supplementary Material provides further information for clarity. Size of blue nodes reflects the number of mir-885 targets it contains. Edges ending in circles (gray) indicate a part of relationship (e.g., FBP1 is a part of the “glycogen storage diseases” pathway, which has the reactome accession number 3229121). Edges ending in straight lines (red) indicate inhibition of the target (e.g., mir-451 inhibits AKT1).

**Figure 3:**
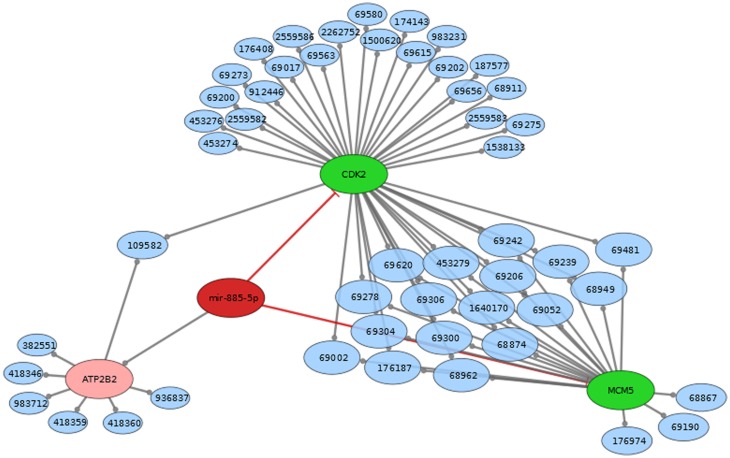
**Mir-885 comes from an intron in ATP2B2 (both red)**. Mir-885 has two known targets (green). These targets are part of a number of reactome pathways (blue). The Number in the blue nodes can be used to access the associated reactome pathway and Table S2 in Supplementary Material provides further information for clarity. Size of blue nodes reflects the number of mir-885 targets it contains. Edges ending in circles (gray) indicate a part of relationship (e.g., CDK2 is a part of the “Activation of the pre-replicative complex” pathway, which has the reactome accession number 68962). Edges ending in straight lines (red) indicate inhibition of the target (e.g., mir-885 inhibits MCM5).

MiRTarBase (23) and TarBase (24) are two databases, which contain miRNA targets with the restriction that each target must have experimental evidence. We therefore used these two databases to determine the targets for construction of a regulatory network intersected with Reactome pathways (25). miR-451 has Anti-Müllerian hormone (*AMH*), Calcium-binding protein 39 (*CAB39*), ATP-binding cassette sub-family B member 1 (*ABCB1*), *AKT1*, IMP2 inner mitochondrial membrane peptidase-like (*IMMP2L*), B-cell lymphoma 2 (*BCL2*), cAMP-regulated phosphoprotein 19 (*ARPP19*), Fructose-1,6-bisphosphatase 1 (*FBP1*), matrix metallopeptidase 9 (*MMP9*), *myc*, transmembrane emp24 protein transport domain containing 7 (*TMED7*), and ubiquitin-conjugating enzyme E2H (*UBE2H*) as confirmed targets. miR-885-5p has the experimentally supported targets cyclin-dependent kinase 2 (*CDK2*), and minichromosome maintenance complex component 5 (*MCM5*). While miR-451 is part of a lincRNA (ENSG00000264066), miR-885 is located in an intron of plasma membrane calcium-transporting ATPase 2 (*ATP2B2*). Overall, miR-451 has 10 targets, which are involved in 154 distinct reactome pathways (Figure 2; Table S2 in Supplementary Material). miR-885-5p has two confirmed targets, which are involved in 50 distinct reactome pathways (Figure 3; Table S3 in Supplementary Material).

### Profiling mRNA expression changes following EPO treatment

We performed high-density oligonucleotide microarray analyses (*n* = 3) to determine the changes in gene expression induced by EPO in SH-SY5Y cells. Our data demonstrate EPO treatment upregulated 51 genes and downregulated 23 genes considering a fold change >1.5 (*p* value <0.05) (Tables 2 and 3).

**Table 2:** **Upregulated genes following 48 h EPO treatment of SH-SY5Y cells**.

Genes	Description	Fold change	*p* value
*DLK1*	Delta, drosophila, homolog like 1	2.645	0.0017
*GAL*	Galanin	2.426	0.0003
*CABP7*	Calcium-binding protein	2.364	4.6E-05
*LOC440063*	Cyclophilin A pseudogene	2.274	0.04
*BHLHB2*	Basic helix loop helix domain containing protein class B2	2.134	0.00025
*IGF2*	Insulin like growth factor 2	2.105	0.00057
*GNAS*	Guanine nucleotide binding protein	2.001	0.00031
*TRK1*	Transfer RNA Lysine 1	1.836	0.030
*NELL2*	NEL like 2	1.828	0.0031
*C20ORF46*	Transmembrane protein	1.813	0.00024
*IGSF21*	Immunoglobulin super family member 21	1.809	0.0032
*RAB6B*	Member RAS oncogene family	1.795	0.00022
*GALNTL4*	UDP-*N*-acetyl-alpha-d-galactosamine:polypeptide; *N*-acetylgalactosaminyltransferase-like 4	1.788	0.00024
*OLFM1*	Olfactomedin 1- neuroblastoma protein	1.78	0.0020
*C20ORF160*	Chromosome 20 open reading frame 160	1.78	0.0036
*SOBP*	Sine Oculus binding protein homolog drosophila	1.766	0.0026
*NR2F2*	Nuclear receptor sub-family 2	1.757	0.0036
*SGPP2*	Sphingosine 1 phosphate phosphatase 2	1.756	0.014
*C3ORF32*	Chromosome 3 open reading frame 32	1.749	0.00046
*SUSD2*	Sushi domain containing 2	1.728	0.0028
*C2CD4B*	C2 Calcium dependent domain containing protein 4B	1.727	0.011
*TSPAN7*	Tetraspanin 7	1.691	0.0008
*VEGFA*	Vascular endothelial growth factor A	1.69	0.0043
*SORBS2*	Sorbin and SH3 domain containing 2	1.686	0.00091
*CNTNAP4*	Contactin associated protein 4	1.662	0.00014
*LOC100132291*	Ribosomal protein S27 pseudogene 29	1.656	0.034
*CDKN1C*	Cyclin-dependent kinase inhibitor 1C	1.651	0.007
*LOC391019*	Ribosomal protein L29 pseudogene 6	1.643	0.023
*LOC729086*	Vesicular overexpressed in cancer, prosurvival protein 1 pseudogene	1.639	0.029
*BOLA2*	Bol A2 homolog (*E. coli*)	1.634	0.0027
*SH2D3C*	SH domain containing protein 3C	1.617	0.0017
*FLJ43681*	Ribosomal protein L23a pseudogene	1.608	0.024
*CYB5A*	Cytochrome b5 type A (microosomal)	1.606	0.002
*LOC442232*	Ribosomal protein L17 pseudogene 25	1.6	0.017
*TSPAN4*	Tetraspanin 4	1.59	0.008
*HES4*	Hairy enhancer of split. drosophila homolog of 4	1.583	0.013
*LOC728820*	Ribosomal protein L29 pseudogene 15	1.582	0.013
*LOC92755*	Tubulin beta pseudogene 1	1.58	0.0063
*CXCR4*	Chemokine CXC motif receptor 4	1.579	0.00029
*HBQ1*	Hemoglobin theta 1	1.575	0.017
*LOC100128060*	Ribosomal protein S7 pseudogene 10	1.562	0.029
*LOC731640*	Ribosomal protein L21 pseudogene 97	1.553	0.016
*AQP10*	Aquaporin 10	1.549	0.0021
*RPL14*	Ribosomal protein L14	1.543	0.033
*SYP*	Synaptophysin	1.541	0.0009
*LOC651453*	Ribosomal protein L36 pseudogene 14	1.53	0.017
*MCM2*	Minichromosome maintenance complex component 2	1.528	0.004
*SLC7A14*	Solute carrier family 7 member 14	1.516	0.028
*INS-IGF2*	INS-IGF2 readthrough	1.513	0.010
*HCN3*	Hyperpolarization activated cyclic nucleotide gated potassium channel 3	1.512	0.0016
*GALNTL1*	UDP-*N*-acetyl-alpha-d-galactosamine:polypeptide; *N*-acetylgalactosaminyltransferase-like 1	1.512	0.008

**Table 3:** **Downregulated genes following 48 h EPO treatment of SH-SY5Y cells**.

Genes	Description	Fold change	*p* value
*FOS*	FBJ murine osteosarcoma viral oncogene homolog	0.172	7.6E-03
*TAGLN*	Transgelin	0.227	6.3E-04
*IL13RA2*	Interleukin 13 receptor, alpha 2	0.344	1.9E-03
*FOSB*	FBJ murine osteosarcoma viral oncogene homolog B	0.344	7.2E-02
*PRSS23*	Protease, serine, 23	0.362	3.5E-03
*MME*	Membrane metallo-endopeptidase	0.382	5.5E-04
*LRRC17*	Leucine rich repeat containing 17	0.41	6.3E-03
*S100A10*	S100 calcium-binding protein A10	0.425	2.9E-04
*TSPAN8*	Tetraspanin 8	0.439	6.0E-03
*EGR1*	Early growth response 1	0.443	5.2E-02
*TP53I3*	Tumor protein p53 inducible protein 3	0.446	3.0E-04
*GPNMB*	Glycoprotein (transmembrane) nmb	0.448	6.3E-03
*SPARC*	Secreted protein, acidic, cysteine-rich	0.455	8.9E-05
*COL3A1*	Collagen, type III, alpha 1	0.457	1.2E-03
*OLFML3*	Olfactomedin-like 3	0.461	2.8E-03
*AHNAK*	AHNAK nucleoprotein	0.467	1.1E-04
*IFITM3*	interferon induced transmembrane protein 3	0.468	1.7E-04
*HTRA1*	HtrA serine peptidase 1	0.476	1.7E-03
*MATN2*	Matrilin 2	0.476	1.7E-02
*COL5A2*	Collagen, type V, alpha	0.489	4.5E-03
*IFITM2*	Interferon induced transmembrane protein 2	0.491	8.8E-04
*MGP*	Matrix Gla Protein	0.492	4.1E-04
*S100A13*	S100 calcium-binding protein A13	0.495	7.5E-02

### qPCR array

**Figure 4:**
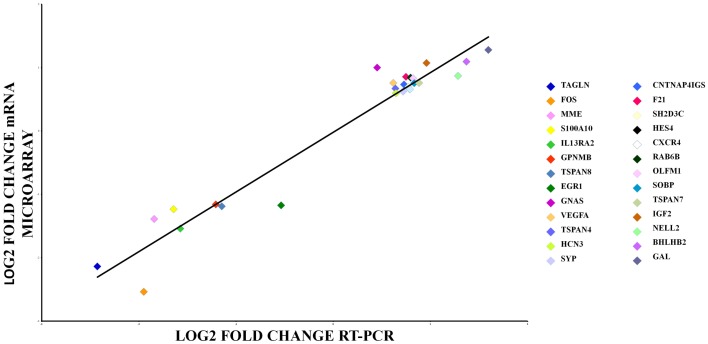
**qRT-PCR array validation of the microarray data for the selected genes**. The RT-PCR data were normalized to the housekeeping gene β-actin. Pearson’s coefficient, *r* = 0.95, *p* = 0.000.

We used qPCR array to validate gene expression changes of 26 genes. Aliquots of the same RNA sample were used for all the experiments in both the microarray and qPCR array measurements. Figure 4 shows that the qPCR findings were highly correlated with the microarray data (Pearson’s *R* = 0.95, *p* = 0.000). Data from the mRNA microarray experiment are shown in Tables 2 and 3 and qPCR results are shown in Tables S4 and S5 in Supplementary Material.

### qPCR analysis of selected target genes at different time point of EPO treatment

**Figure 5:**
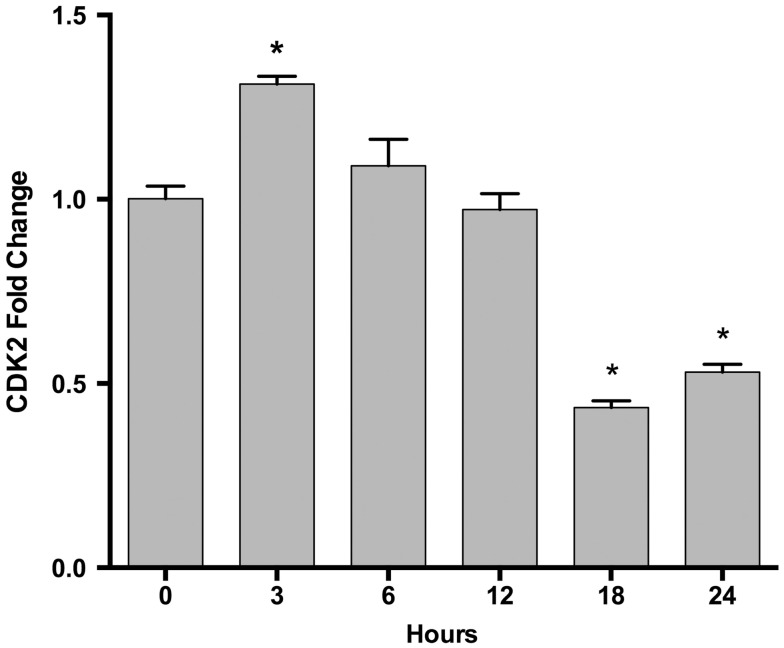
**Time-dependent CDK2 gene expression changes following EPO treatment in SH-SY5Y cells**. Cells were treated with 1 U/ml EPO during 0–24 h time intervals. Expression levels of CDK2 were quantified by qRT-PCR. CDK2; cell cycle dependent kinase 2. The data are presented as mean ± SE, *n* = 5 (*p < 0.05).

We examined the gene expression levels of these miRNA target genes at different time points after EPO treatment. We found that EPO upregulates matrix metallo peptidase 9 (*MMP9)*, B-celllymphoma 2 (*BCL2), EPOR*, and Galanin (*GAL)* expression at 24 and 48 h, vascular endothelial growth factor A (*VEGFA)*, mini chromosome maintenance complex 5 (*MCM5)* at 48 h, and cyclin-dependent kinase 2 (*CDK2)* expression at 3 h (Table 4; Figure 5).

**Table 4:** **Selected target mRNA expression changes of miR-451 and miR-885-5p in EPO-treated SH-SY5Y cells**.

MicroRNA	Target mRNA	24 h EPO treatment		48 h EPO treatment	
		Fold change	*p* Value	Fold change	*p* Value
miR-451	*BCL2*	2.19	0.014	2.52	0.014
	*VEGFA*	1.01	0.917	1.59	0.014
	*EPOR*	0.97	0.917	1.48	0.009
miR-885-5p	*MCM5*	1.02	0.917	1.56	0.009
	*GAL*	0.98	0.806	1.73	0.014
Both (miR-451 and miR-885-5p)	*MMP9*	1.44	0.028	2.10	0.009

### Upregulation of miR-451 and miR-885-5p expression can inhibit their target gene expression

**Figure 6:**
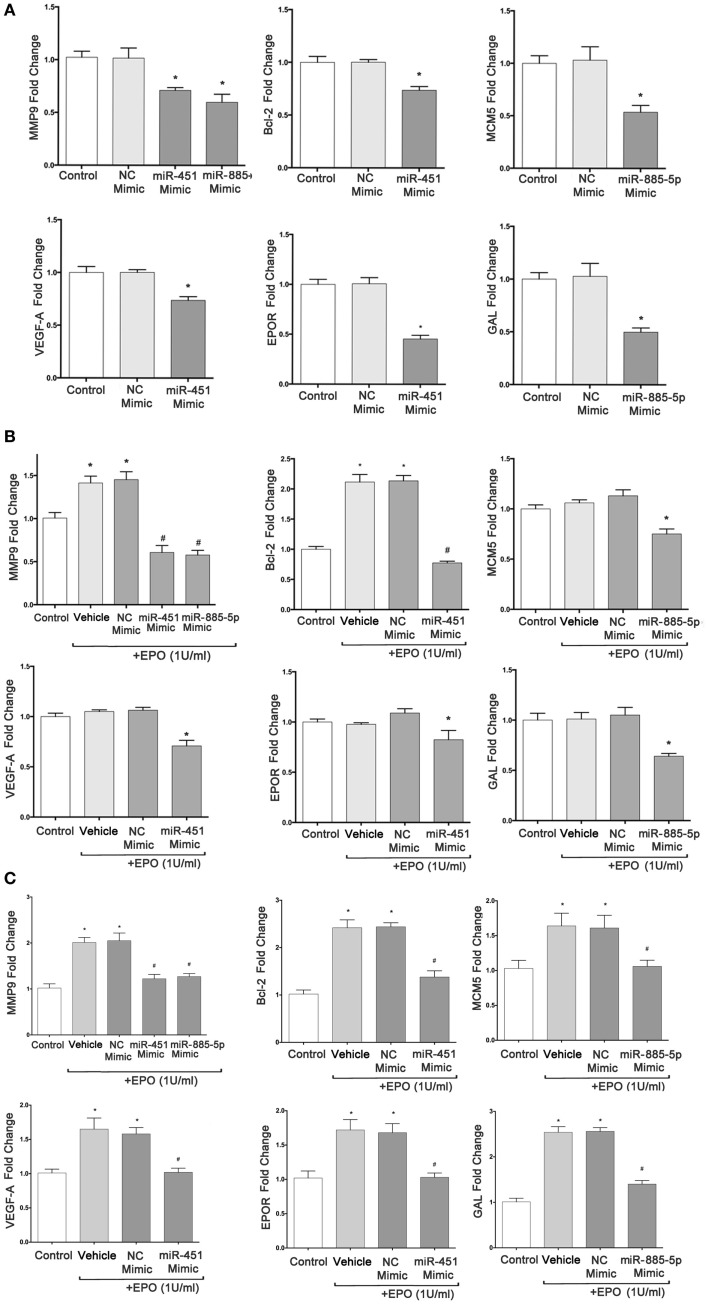
**miR-451 and miR-885-5p significantly downregulate expression of target genes in SH-SY5Y cells**. qRT-PCR was performed to determine the mRNA levels of target genes after transfection of SH-SY5Y cells with miR-451, miR-885-5p mimics, and negative control (NC) miRNAs without EPO treatment **(A)**, with 24 h EPO treatment **(B)** and 48 h EPO treatment **(C)**. Control: cells without any transfection. The data are presented as mean ± SE, *n* = 5 (*p < 0.05).

To determine whether differentially expressed miRNA can regulate their target genes’ expression in SH-SY5Y cells, we investigated the modulation of *MMP9, BCL2, VEGFA, EPOR, MCM5*, and *GAL* mRNA levels in cells transfected with mimics of miR-451 and miR-885-5p by qPCR. The result of real-time qPCR revealed that miR-451 and miR-885-5p mimics can significantly decrease the basal expression of *MMP9, BCL2, VEGFA, EPOR, MCM5*, and *GAL*, suggesting that mimics efficiently introduced into the cells and decreased the expression of their target genes (Figure 6A).

We found that cells transiently transfected with miR-451 and miR-885-5p mimics had significantly reduced EPO-induced target genes expression at 24 h *(MMP9, BCL2)* and at 48 h *(MMP9, BCL2, VEGFA, EPOR, MCM5*, and *GAL)* (Figures 6B,C).

### Downregulation of miR-451 and miR-885-5p contributes to the neuroprotective effects of EPO

**Figure 7:**
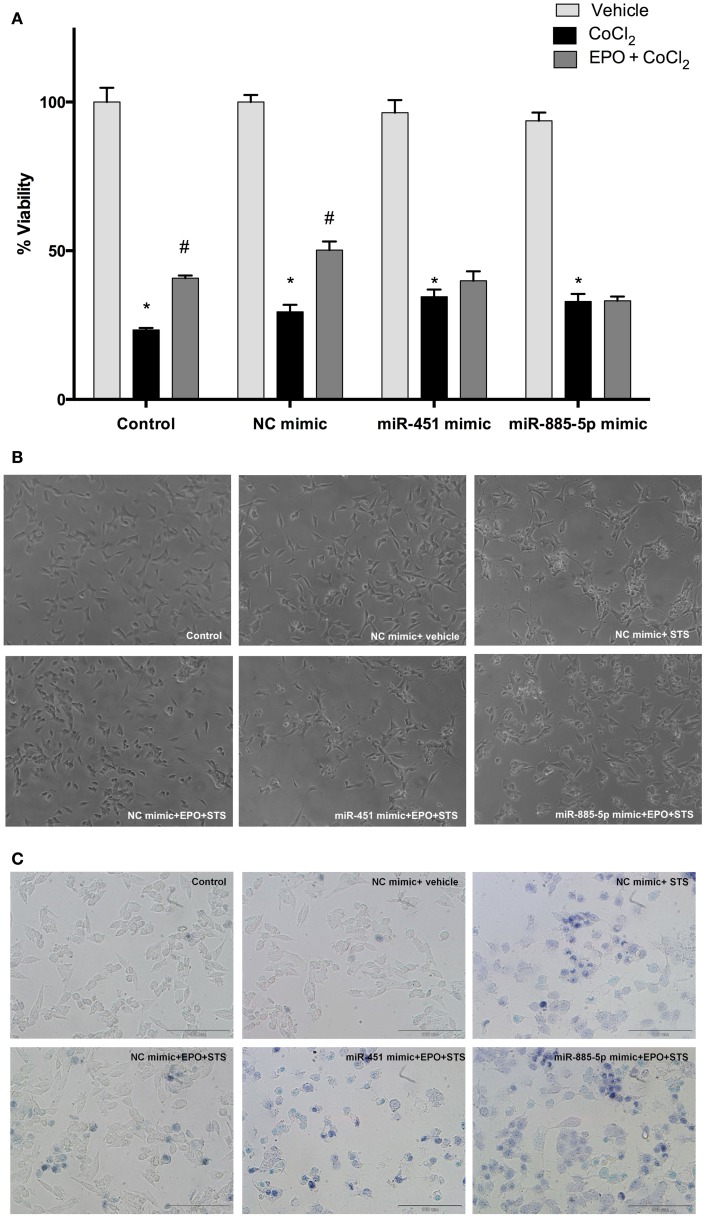
**miR-451 and miR-885-5p may mediate the neuroprotective effect of EPO**. Transfected and non-transfected cells were treated with EPO and/or CoCl_2_. **(A)** Cell viability was analyzed by WST-8 assay, **(B)** representative phase-contrast microcopy images of SH-SY5Y cells, and **(C)** representative light microscopy images showing trypan blue stained dead cells. Over-expression of miR-451 and miR-885-5p reverse the neuroprotective effect of EPO induced by CoCl_2_. CoCl_2_; cobalt chloride, EPO; erythropoietin. The data are presented as mean ± SE, *n* = 5 (*p < 0.05 compared to control and ^#^*p* < 0.05 compared to CoCl_2_ treated cells).

To evaluate the role of miR-451 and miR-885-5p in the protective effect of EPO, SH-SY5Y cells were transfected with mimics to investigate the effect of over-expression of these miRNAs. The EPO responsiveness in respect to cell death induced by CoCl_2_ was examined with the WST-8 assay. Cell viability significantly decreased with CoCl_2_, which can be reversed by EPO pretreatment. After upregulation of miR-451 and miR-885-5p levels with mimics, the cell viability levels in EPO-treated cells did not reach the levels in the negative control mimic group (Figures 7A–C). This finding suggests that the protective effect of EPO was in part dependent on the expression levels of miR-451 and miR-885-5p.

### Over-expression of miR-451 and miR-885-5p can reverse anti-apoptotic effect of EPO

Previous findings indicated that EPO inhibits apoptosis induced by STS. Here, we investigated the effects of EPO on apoptosis of SH-SY5Y cells following an increase in miR-451 and miR-885-5p levels upon transfection with mimics.

**Figure 8:**
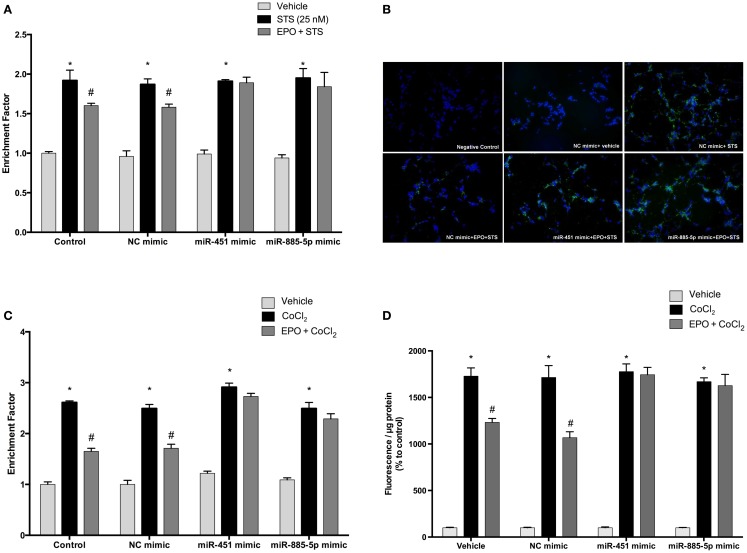
**Anti-apoptotic and anti-oxidant effects of EPO in SH-SY5Y cells may be regulated by miR-451 and miR-885-5p**. Transfected and non-transfected cells were treated by staurosporine (STS) (25 nM) and 1 U/ml EPO. **(A)** DNA fragmentation was evaluated by cell death ELISA assay. **(B)** Apoptotic phosphatidylserine (PS) positive cells were stained by Annexin-V-FITC dye and visualized using immunofluorescence microscopy. Cells were also treated with CoCl_2_ (250 μM) and 1 U/ml EPO **(C)** and DNA fragmentation was analyzed by cell death ELISA assay. Transfection of miR-451 and miR-885-5p mimics reduced the anti-apoptotic effect of EPO in both STS and CoCl_2_ induced apoptosis. **(D)** Intracellular ROS production was quantified by using the CM-H_2_DCFDA method. Over-expression of miR-451 and miR-885-5p reversed anti-oxidant effect of EPO induced by CoCl_2._ STS; staurosporin, EPO; erythropoietin, CM-H_2_DCFDA; 2′,7′-dichlorodihydrofluorescein, acetyl ester. The data are presented as mean ± SE, *n* = 5 [*p < 0.05 compared to control and ^#^*p* < 0.05 compared to toxic agents (STS or CoCl_2_) treated cells].

Cells were transfected with mimics to simulate over-expression of miR-451 and miR-885-5p. As expected, EPO effects on miR-451 and miR-885-5p expression were diminished in cells transfected with mimics (Figures 8A,B). Consequently, EPO-mediated anti-apoptotic effect was significantly reduced in the mimics transfected cells as compared with cells that were transfected with control mimics. This indicates the importance of miR-451 and miR-885-5p in mediating the anti-apoptotic effect of EPO. We also found that there is an impact of miR-451 and miR-885-5p on EPO’s anti-apoptotic effects in response to CoCl_2_ induced apoptosis (Figure 8C).

### Over-expression of miR-451 and miR-885-5p can reverse the anti-oxidant effect of EPO

In order to evaluate the role of miR-451 and miR-885-5p for the anti-oxidant effect of EPO, we investigated the effects of EPO on ROS production induced by CoCl_2_ in SH-SY5Y cells after transfection with mimics. As shown in Figure 8D, the anti-oxidant effect of EPO was attenuated by mimics of miR-451 and miR-885-5p. Our results indicate the importance of miR-451 and miR-885-5p in mediating the anti-oxidant effect of EPO.

### Upregulation of miR-451 and miR-885-5p affects proliferation of SH-SY5Y cells enhanced by EPO

**Figure 9:**
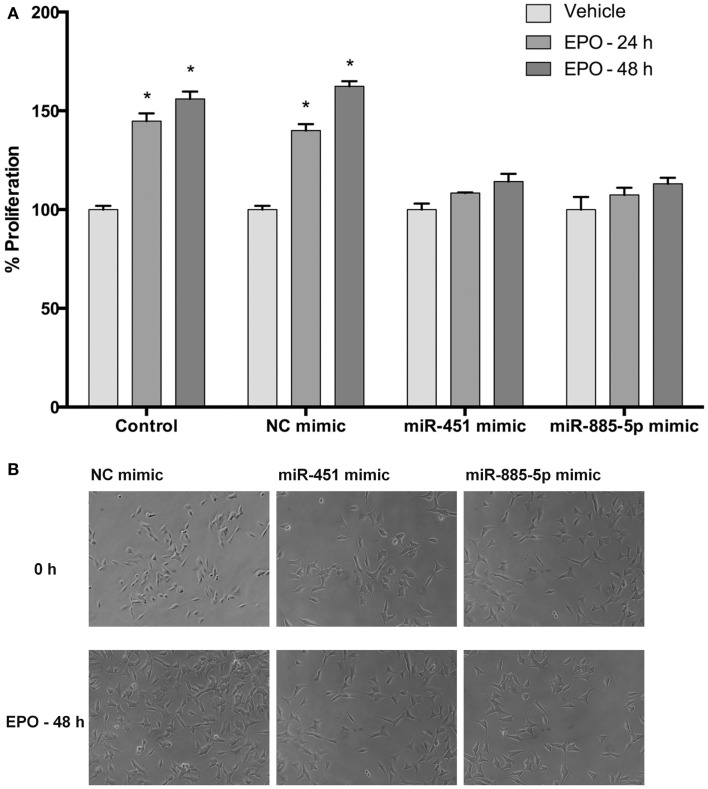
**Upregulation of miR-451 and miR-885-5p reduces proliferation of SH-SY5Y cells enhanced by EPO**. In miRNA mimic transfected and non-transfected cells, cell proliferation was observed by counting cells using phase-contrast microscopy following either 24 or 48 h EPO treatment. **(A)** Cell counts and **(B)** phase-contrast microscopy images. Transfection of miR-451 and miR-885-5p mimics reduces the proliferative effect of EPO. EPO; erythropoietin. The data are presented as mean ± SE, *n* = 5 (*p < 0.05).

Cell proliferation was evaluated after transfection with mimics to determine the impact of miR-451 and miR-885-5p on the proliferative effect of EPO. According to our results, EPO increases proliferation of SH-SY5Y cells at 24 and 48 h after treatment (Figures 9A,B). We found that cells transiently transfected with miR-451 and miR-885-5p mimics had significantly reduced proliferation as compared with cells transfected with control mimics.

### Downregulation of miR-451 and miR-885-5p contributes to the neuronal migratory effect of EPO

**Figure 10:**
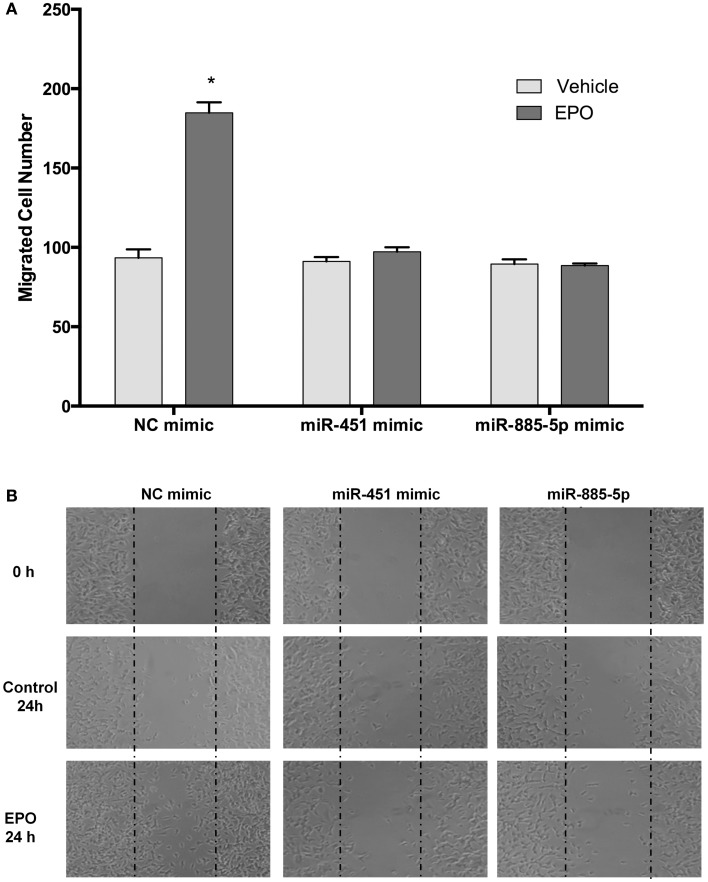
**EPO induces migration in SH-SY5Y cells, which can be reversed by mir-451 and mir-885-5p**. Transfected and non-transfected cells were treated with 1 U/ml EPO and migration assay was conducted for either condition. **(A)** Cells were counted by using ImageJ 1.42 (http://imagej.nih.gov/ij/). **(B)** Representative images of migrated SH-SY5Y cells. Over-expression of miR-451 and miR-885-5p reverse the migratory effect of EPO. EPO, erythropoietin. The data are presented as mean ± SE, *n* = 3 (*p < 0.05).

We further investigated the role of miR-451 and miR-885-5p on the migratory effects of EPO. SH-SY5Y cells were transfected with miR-451 and miR-885-5p mimics or control miRNAs for 48 h and then EPO responsiveness on cell migration was examined with scratch assay. We found that the EPO-mediated migratory effect was significantly reduced in the mimics transfected cells as compared with cells that were transfected with control mimics (Figures 10A,B).

### Effects of miR-451 and miR-885-5p upregulation on EPO-mediated neurite outgrowth and neurite length in SH-SY5Y cells

**Figure 11:**
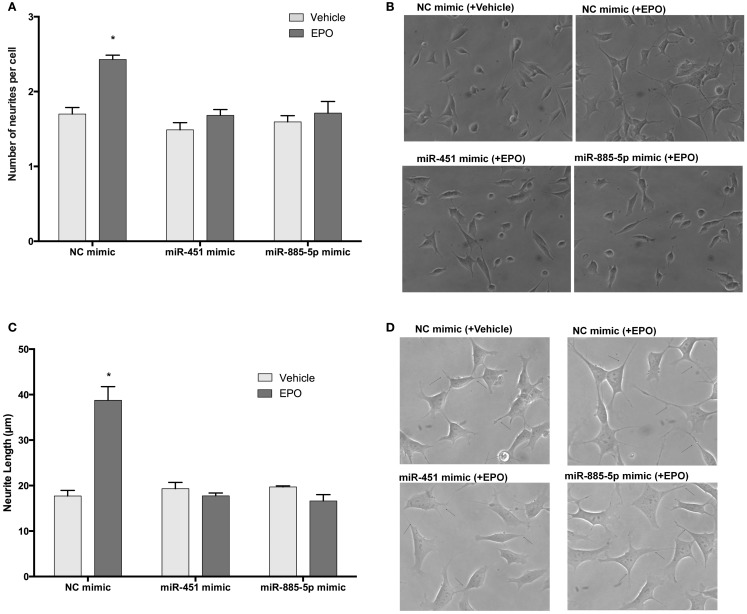
**EPO may enhance neurite outgrowth and length via regulation of miR-451 and miR-885-5p**. Transfected and non-transfected cells were treated with 1 U/ml EPO. **(A)** Neurite cell number was counted using ImageJ 1.42 (http://imagej.nih.gov/ij/). Randomly selected fields in microscopic analysis with an average of 35 cells per field were analyzed. **(B)** Representative phase-contrast microscopy images are shown in order to indicate the increased neurite numbers induced by EPO and reversed by the transfection of mimics of miR-451 and miR-885-5p. **(C)** The average maximal neurite length for both, the transfected and non-transfected cells were analyzed by ImageJ 1.42 (http://imagej.nih.gov/ij/). At least 30 neurites per condition were measured. **(D)** Neurite length distribution. Arrowheads indicate the neurites arising from cells. Scale bar, 100 μm. EPO, erythropoietin. The data are presented as mean ± SE, *n* = 5 (*p < 0.05).

To investigate the role of miR-451 and miR-885-5p upregulation on EPO-mediated neurite outgrowth in SH-SY5Y cells, we used mimics to simulate over-expression of miR-451 and miR-885-5p. EPO significantly increased neurite outgrowth to 69.5 ± 5.3% relative to controls and the increase remained unchanged in negative control mimics transfected cells (73.4 ± 3.9%). However, the increase due to EPO treatment in the percentage of neurite-bearing cells was significantly attenuated in those cells transfected with mimics (Figures 11A,B).

Similar results were obtained for neurite length analysis. EPO increases neurite length of SH-SY5Y cells after 24 h treatment (Figures 11C,D). We found that cells transiently transfected with miR-451 and miR-885-5p mimics had significantly reduced neurite length compared with that of cells transfected with control mimics. These results show that the miR-451 and miR-885-5p are involved in the induction of neurite outgrowth in SH-SY5Ycells.

## Discussion

Erythropoietin is a well-known cytokine growth factor, which exerts pleiotropic effects including neurotrophic, neuroprotective, anti-apoptotic, anti-oxidative, and immune-modulatory activities. For instance, EPO inhibits pro-inflammatory cytokine release and T cell responses in experimental autoimmune encephalomyelitis (EAE) (26, 27). Proposed mechanisms of neuroprotective effects of EPO include gene expression regulation at the post-transcriptional level including alteration of miRNA expression. Here, we analyzed the effect of EPO on miRNA expression in SH-SY5Y neuronal-like cells. MicroRNA microarray experiments showed that miR-451 and miR-885-5p were downregulated by 24 h EPO treatment. Real-time qPCR studies verified the miRNA microarray results.

In this *in vitro* study, we chose SH-SY5Y human neuroblastoma cells as cell culture model because of their high transfection efficiency. SH-SY5Y cells express EPOR and other tissue-protective EPO receptors such as CD131 (26). In addition, cellular and molecular actions of EPO have been widely studied in this model such as proliferative, anti-apoptotic, anti-oxidant, anti-inflammatory, and neurite outgrowth promoting effects (28–30).

In this study, we demonstrated, for the first time, that select miRNAs are regulated by EPO in neuronal SH-SY5Y cells. Previously, only one study evaluated the effect of EPO on the miRNA expression profile in murine erythroid cells (18). In that study, miR-210, miR-188, and miR-362 were significantly elevated with stimulation of EPO. miR-210 has been demonstrated to be a neuroprotective miRNA against oxygen-glucose deprivation in PC12 cells (31). This miRNA is well known to stimulate angiogenesis and neurogenesis (32). As activity-regulated miRNA, miR-188 shows neurotrophic effects in rat hippocampal neurons (33). We could not detect any change in miR-210 and miR-188 expression profiles in EPO-treated SH-SY5Y cells. This discrepancy may be due to species and cell type differences. Another plausible explanation could be the use of different exposure duration and dose of EPO. In our hand, EPO dose of one unit did not show any change in the expression of miR-210 in time course qPCR experiments (3–48 h, data not shown). Our ongoing studies, especially, focus on whether miR-210 mediates the promoting effect of EPO on angiogenesis and neurogenesis in neural progenitor and cerebral endothelial cells.

We performed mRNA microarray analysis to evaluate mRNA changes in EPO-treated SH-SY5Y cells. EPO treatment in SH-SY5Y neuronal cells upregulated 51 genes and downregulated 23 genes. EPO caused changes in the expression of genes involved in cell proliferation, cell survival, and cell differentiation. There are some contradictory and similar results as compared with previous EPO mRNA array studies in the literature. The discrepancies that discussed above in detail may be due to technical limitations that also interfere with miRNA expression profiling results. Although the comparison of *in vitro* and *in vivo* findings may be somewhat problematic, the results of a previous *in vivo* study by Anderson et al. are comparable with our transcriptome profiling (14). They found that chemokine (C–X–C Motif) receptor 4 (*CXCR4), Gal*, and *IGF2* upregulated at different time points, 24 h–7 days, in the brains of EPO-treated rats. In another experimental rodent infarct model, CXCR4 was found significantly upregulated in EPO-treated rats (34). Although *VEGFA* upregulation has not been reported in previous mRNA array studies, we found that EPO increased *VEGFA* expression by qPCR. As a neuroprotectant, neurotrophic, and angiogenic molecule, VEGFA may mediate the effects of EPO in the nervous system (35). Conversely, EPO-mediated *VEGFA* gene regulation may proceed via downregulation of miR-451, as revealed by our mimic transfection study. *Gal*, which is a target of miR-885-5p has neuroprotective, neurotrophic, and immunomodulatory effects (36). Future studies using *Gal* siRNA or GAL receptor blockers may delineate the *Gal* gene regulation mechanisms induced by EPO. Using cultured rat PC12 cells or rat B104 neuroblastoma cells, other groups found upregulation in anti-apoptotic genes, *BCLXL* and *BCL2*, respectively (9, 37). We also found an increase in *BCL2* expression by qPCR. We also confirmed the finding that EPO-mediated *EPOR* expression in SH-SY5Y cells (28, 38). In addition, we also studied cell cycle regulating genes, *MCM5* and *CDK2* that are targets of miR-885-5p (39). EPO significantly increased their expression and mimics of miR-885-5p reversed the resulting effect, suggesting that EPO regulates cell cycle genes in an miRNA-dependent manner. As validated targets of EPO downregulated miRNAs (miR-451 and miR-885-5p), MMP9 may mediate neuronal migratory and angiogenic effects of EPO.

In the current study, we choose a single time point for mRNA assay. In the literature, some EPO-induced genes such as *BCLXL are* upregulated as early as at 3 h and the expression of others peaked at a very late time point such as 7 days (*MMP9*) (9, 14). Thus, we might fail to detect possible expression changes of some EPO-regulated genes such as *CDK2*. As a powerful technique and gold standard method, qPCR may detect tiny expression changes as revealed in our study. In contrast to mRNA array, qPCR showed upregulation of some EPO-regulated genes (*BCL2, EPOR*, and *MCM5*) at 48 h.

We found some genes whose expressions are upregulated by EPO in our mRNA microarray study: *IGF2* (neuroprotection), *CXCR4* (neural migration, immunomodulation, neuroprotection), and *synaptophysin* (synaptogenesis). Since differentially regulated miRNAs, miR-451 and miR-885-5p, did not target those genes, we did not focus on miRNA-mediated regulation of them in this study. Future studies may provide insight on the roles of these miRNA–mRNA interactions on the effects of EPO. As shown in non-neuronal cells, miRNAs may be both regulators and mediators of EPO’s action. For instance, miR-125b downregulates EPO and EPOR expression in breast MCF7 cancer cell lines (40). In another study, brain enriched miRNA, miR-132 downregulated the expression of EPO via the inhibition of Nrf2 transcription factor in rat epithelial cells (41).

In this study, we did not focus on the possible mechanism of EPO-mediated miRNA regulation. EPO modulates various signaling pathways and transcription factors and may use these mechanisms to regulate miRNA expression (8). Another interesting point is the differences between EPO and its variant carbamylated EPO (42). First, the effects of EPO variants on miRNA expression profiling should be studied. Then supporting functional studies should be performed using mRNAs mimics and antagomirs. EPO exerts similar cytoprotective effects in non-neuronal cells of nervous system. Thus, the results of miRNA expression profiling studies using cultured astrocytes, microglia, oligodendroglia, and cerebral endothelial cells will also be informative. Here, the expression of pri- and pre-forms of miRNAs was not studied and only mature forms were evaluated. Such studies will give more information on the molecular level(s) in which EPO modulates specific miRNAs. Another issue that should be addressed in future studies is the detection of exosomal miRNAs, especially miR-451, in the EPO-treated supernatant of cultured cells (43, 44).

The extrapolation of *in vitro* results to *in vivo* status is not without limitation. Thus, experimental animal studies should also be designed and performed using mimics or antagomirs of selected miRNAs. Complementary behavioral, electrophysiological, and bioinformatics analyses are also a mandatory part of *in vivo* studies combined miRNAs *in situ* hybridization/immunohistochemical analyses or sophisticated techniques such as laser captured microdissection microscopy will help to reveal miRNAs expression changes at the cellular level.

In conclusion, to the best of our knowledge, this is the first integrative miRNA–mRNA study using EPO. Such studies will uncover the mechanism of cellular and molecular actions of EPO at different regulation steps. Future studies focusing on miRNA-mediated mechanisms may provide novel insight on how biological effects of EPO are modulated.

## Conflict of Interest Statement

The authors declare that the research was conducted in the absence of any commercial or financial relationships that could be construed as a potential conflict of interest.

## Supplementary Material

The Supplementary Material for this article can be found online at http://www.frontiersin.org/Journal/10.3389/fimmu.2014.00475/abstract

## Footnotes

^1^http://www.umm.uni-heidelberg.de/apps/zmf/mirwalk/

^2^http://www.reactome.org/

^3^http://mirtarbase.mbc.nctu.edu.tw/

^4^http://diana.imis.athena-innovation.gr/DianaTools/index.php?r=tarbase/index

^5^http://imagej.nih.gov/ij/
