# Supplementary material for: EPO Mediates Neurotrophic, Neuroprotective, Anti-Oxidant, and Anti-Apoptotic Effects via Downregulation of miR-451 and miR-885-5p in SH-SY5Y Neuron-Like Cells
Source: Front Immunol. 2014 Sep 30;5:475. doi: 10.3389/fimmu.2014.00475 (PMC4179732; doi:10.3389/fimmu.2014.00475)
Supplement: Supplementary file 4 [file Table4.DOCX]

**Table S4. Upregulated genes in qPCR array following 48 hours EPO treatment of SH-SY5Y cells**

| **Genes** | **Description** | **Fold Change** | ***p* value** |
| --- | --- | --- | --- |
| **GAL** | Galanin | 3,023 | 3,49071E-05 |
| **BHLHB2** | Basic helix loop helix domain containing protein class B 2 | 2,584 | 0,007 |
| **NELL2** | NEL like 2 | 2,435 | 0,00021 |
| **IGF2** | Insulin like growth factor 2 | 1,945 | 0,0049 |
| **TSPAN7** | Tetraspanin 7 | 1,843 | 0,0013 |
| **SOBP** | Sine Oculus binding protein homolog drosophila | 1,781 | 0,00048 |
| **OLFM1** | Olfactomedin 1- Neuroblastoma proteini | 1,766 | 0,0019 |
| **RAB6B** | Member RAS oncogene family | 1,735 | 0,0015 |
| **CXCR4** | Chemokine CXC motif reseptor 4 | 1,728 | 0,0018 |
| **HES4** | Hairy enhancer of split, drosophila homolog of 4 | 1,721 | 0,025 |
| **SH2D3C** | SH domain containing protein 3C | 1,707 | 0,00015 |
| **IGSF21** | Immunoglobulin super family member 21 | 1,680 | 0,00025 |
| **CNTNAP4** | Contactin associated protein 4 | 1,655 | 0,0119 |
| **SYP** | Synaptophysin | 1,645 | 0,0021 |
| **HCN3** | Hyperpolarization activated cyclic nucleotide gated potassium channel 3 | 1,563 | 0,0030 |
| **TSPAN4** | Tetraspanin 4 | 1,558 | 0,00021 |
| **VEGFA** | Vascular endothelial growth factor A | 1,534 | 0,00071 |
| **GNAS** | Guanine nucleotide binding protein | 1,368 | 0,0026 |
