# Supplementary material for: EPO Mediates Neurotrophic, Neuroprotective, Anti-Oxidant, and Anti-Apoptotic Effects via Downregulation of miR-451 and miR-885-5p in SH-SY5Y Neuron-Like Cells
Source: Front Immunol. 2014 Sep 30;5:475. doi: 10.3389/fimmu.2014.00475 (PMC4179732; doi:10.3389/fimmu.2014.00475)
Supplement: Supplementary file 5 [file Table5.DOCX]

**Table S5. Downregulated genes in qPCR array following 48 hours EPO treatment of SH-SY5Y cells**

| **Genes** | **Description** | **Fold Change** | ***p* value** |
| --- | --- | --- | --- |
| **EGR1** | Early growth response 1 | 0,689 | 0,060 |
| **TSPAN8** | Tetraspanin 8 | 0,451 | 0,00030 |
| **GPNMB** | Glycoprotein (transmembrane) nmb | 0,433 | 0,00011 |
| **IL13RA2** | Interleukin 13 receptor, alpha 2 | 0,336 | 6,2647E-06 |
| **S100A10** | S100 calcium binding protein A10 | 0,320 | 0,0083 |
| **MME** | Membrane metallo-endopeptidase | 0,279 | 0,00088 |
| **FOS** | FBJ murine osteosarcoma viral oncogene homolog | 0,259 | 0,0040 |
| **TAGLN** | Transgelin | 0,186 | 4,0895E-05 |
